# Analysis of Psychological Well-Being from a Compositional Data Analysis Perspective: A New Approach

**DOI:** 10.3390/bs13110926

**Published:** 2023-11-13

**Authors:** María Cortés-Rodríguez, Purificación Galindo-Villardón, Mercedes Sánchez-Barba, Eusebi Jarauta-Bragulat, José David Urchaga-Litago

**Affiliations:** 1 Department of Statistics, University of Salamanca, 37008 Salamanca, Spain; pgalindo@usal.es (P.G.-V.); mersanbar@usal.es (M.S.-B.); 2 Department of Hematology, Hospital Universitario de Salamanca, Institute for Biomedical Research of Salamanca (IBSAL), 37007 Salamanca, Spain; 3 Departament of Psychology, Pontifical University of Comillas, 28015 Madrid, Spain; 4 Centro de Estudios e Investigaciones Estadísticas, Escuela Superior Politécnica del Litoral (ESPOL), Campus Gustavo Galindo, Guayaquil 090112, Ecuador; 5 Centro de Estudios Estadísticos, Universidad Estatal de Milagro, Milagro 091050, Ecuador; 6 Department of Civil and Environmental Engineering, North Campus, Technical University of Catalonia, 08034 Barcelona, Spain; eusebi.jarauta@upc.edu; 7 Faculty of Communication, Pontifical University of Salamanca, 37002 Salamanca, Spain; jdurchagali@upsa.es

**Keywords:** compositional data analysis, psychological well-being, log ratio general indicator

## Abstract

Well-being is a widely studied construct in psychology. In 1989, Carol Ryff proposed the “Scale of Psychological Well-Being (SPWB)”, which has been validated in multiple languages. The instrument assesses six dimensions of psychological well-being: Self-acceptance, Positive Relationships with Others, Autonomy, Environmental Mastery, Purpose in Life, and Personal Growth. In this article, we propose to enrich the traditional approach of directly interpreting the raw scores in each dimension by incorporating Compositional Data Analysis. This new approach aims to identify “what proportion” of each dimension constitutes well-being, which will allow us to analyze the interactions between the different dimensions of well-being and balance among them. To achieve this, we introduce two position ratios (PR1 and PR2) and a general adjustment indicator called the General Indicator of Subjective Psychological Well-Being (GISPW), which characterizes individuals in a compositional manner, providing a fresh perspective in the interpretation of psychological test results, specifically those related to PWB. The proposal is illustrated with three cases taken from a study involving 628 university students who completed the psychological well-being scale questionnaire. The results show that the GISPW, PR1, and PR2 obtained offer relevant information about the overall balance of each case in the different dimensions.

## 1. Introduction

Well-being can be defined from two different perspectives: as a concept related to individual happiness, in which case we refer to subjective well-being (SWB) [1], or as a concept linked to the development of human potential, in which case it is referred to as psychological well-being (PWB) [2]. This work focuses on the latter approach, specifically studying PWB as the positive functioning of the individual in various areas of life, since the scale under study in this work is based on this second approach. The study of well-being and its assessment is of vital importance for healthcare professionals, as well-being is a fundamental component of overall health [3]. The study of psychological well-being is useful for the early detection of mental or emotional disorders, as well as for the recovery of hospitalized individuals [4]. Well-being allows for the identification of risk factors that guide early intervention [5,6].

In 1989, Carol Ryff introduced an instrument for measuring psychological well-being. Before this, there had been attempts to measure well-being, such as Diener’s Satisfaction with Life Scale (SWLS) [7], Cantril’s Well-being Scale [8], or Bradburn’s Affect Balance Scale [9]. However, all these instruments focused on specific aspects of well-being. The originality of Ryff’s scale lies in its multidimensional approach, which includes six dimensions: Positive Relations with Others (PRE), Autonomy (AUT), Environmental Mastery (CEN), Personal Growth (PGR), Purpose in Life (PLI), and Self-acceptance (SAC), providing a holistic evaluation of the well-being concept. Each of these dimensions are described below.

Self-acceptance: This dimension refers to the ability to have a positive attitude towards and acceptance of oneself, including both strengths and weaknesses. It involves the recognition and appreciation of one’s own identity and healthy self-esteem. Positive Relations with Others: This dimension focuses on the quality of interpersonal relationships. It implies having gratifying and healthy relationships with friends, family, and the community at large. Positive social connections and emotional support are essential components of this dimension. Autonomy: Autonomy refers to the ability to make decisions and act according to one’s own values and goals. It implies having a sense of independence and self-direction, and not being overly influenced by external expectations or pressures. Mastery of the Environment: This dimension relates to the perception of competence and effectiveness in facing the challenges and demands of the environment. It includes having problem-solving skills, adaptability, and confidence in one’s own abilities to overcome obstacles. Purpose in Life: This refers to having a sense of direction and meaning in life. It implies having clear goals and motivations, and feeling committed to something larger than oneself. This dimension is associated with the feeling that life has a purpose and a sense of personal transcendence. Personal Growth: This dimension involves the desire to seek self-development and self-realization throughout life. It refers to being open to change, learning from experiences, seeking new growth opportunities, and developing personal potential.

To develop the questionnaire, Ryff [10] based her ideas on the existing literature, integrating theories from the fields of mental health and humanistic psychology. The interpretation of scores is straightforward; for instance, higher scores in the “Personal Growth” dimension suggest that an individual puts more effort into developing their potential, into continuing their Personal Growth, and maximizing their abilities. Based on the individual’s scores across the different factors, a higher or lower level of psychological well-being can be established.

The statistical processing carried out so far has generally involved the separate analysis of each factor of PWB or aggregated individually, but the relative weight of each factor in relation to the whole has not been studied. This assumes that, for example, up until now, if a person on a health scale with three factors whose scores ranged from 0 to 70 obtained the scores F1: 10, F2: 20, and F3: 70, the overall health score would be 100 points. In compositional data, the first thing of interest is to know the weight of each factor in one’s health, such that factor 1 represents 10% of their health, factor 2 represents 20%, and factor 3 represents 70%. It is possible to study the imbalance/balance between these factors, so one subject (case A) may have high scores and a large imbalance (F1: 20, F2: 70, F3: 50), while another subject (case B) may have high scores but with a great balance (F1: 50, F2: 50, F3: 40). On the other hand, we could have a subject (case C) with low scores (F1: 10, F2: 35, F3: 25), but who would obtain the same profile as subject A (F1: 14.28%, F2: 50%, F3: 35.72%).

This article proposes a different and novel approach in which the individual’s perception of psychological well-being, as manifested in their response to the questionnaire, is considered as a whole, and the elements of PWB form parts of this whole. For this purpose, a different statistical methodology based on the concepts and methods of Compositional Data Analysis [11,12,13] is proposed.

Statistical analysis of compositional data has its origins in various problems encountered in geology [14] and health sciences [15], where proportions are used, leading to challenges when working with matrices and correlation coefficients. Aitchison [11] addressed this problem by defining a new geometry, the simplex geometry, for compositional data. The objective of statistical analysis of compositional data in this context is to analyze the relative distribution of different dimensions within a construct in relation to the whole.

In the field of human food sciences, Compositional Data Analysis has recently been applied successfully [16], and in the domain of psychological tests, the first proposal incorporating it has been published [17]. Traditionally, to interpret a psychological test, the sum of the items for each dimension and the overall construct are calculated, and these scores are then interpreted. However, for PWB, there are no chronological measurements, percentiles, or standardized scores to help interpret the questionnaire results. Interpretation is typically based on the minimum or maximum possible score on that particular scale.

Compositional data have the fundamental characteristic that the sum of all values from the different dimensions of the same individual results in a constant (usually 1 or 100%), and the individual’s value in each dimension is not considered in isolation. Instead, it is evaluated in relation to the total and, therefore, in relation to the other dimensions, providing a reference to interpret the scores of individuals.

The objective is to exemplify in what proportion the different dimensions of psychological well-being explain this well-being in different specific cases.

To explain the relative position of an individual’s dimensions with respect to a general centrality indicator on the one hand and, on the other hand, in relation to a dimension-specific centrality indicator.

We aimed to elucidate how Compositional Data Analysis can be applied to profiles of both groups and specific cases.

## 2. Materials and Methods

### 2.1. Procedure and Participants

A descriptive cross-sectional study was conducted with the aim of examining the psychological well-being among university students. The sample consisted of 628 Spanish students, of which 74% were female. The participants’ ages ranged from 18 to 24 years, with a median age of 19 years.

To collect the data, a mixed-method approach was employed, and the sampling method used was non-probabilistic convenience sampling. The survey was distributed both online through social media and in physical format in different faculties of the University of Salamanca. Precautions were taken to ensure data confidentiality, and participants were informed about the research purpose and the protection of their privacy. All participants provided their consent to respond to the survey.

### 2.2. Research Instruments

Carol Ryff’s Psychological Well-being Scale, adapted by Diaz et al. [18], was used in this study, consisting of six dimensions (PRE, AUT, CEN, PGR, PLI, and SAC), each with four to six items. The scale consists of 29 questions with six response options, ranging from strongly disagree to strongly agree. The response scale has six options with scores ranging from 1 (completely disagree) to 6 (completely agree). A higher score in each dimension indicates a greater degree of well-being. The scale demonstrates good internal consistency with Cronbach’s α scores above 0.7 (PRE: 0.78, AUT: 0.7, CEN: 0.82, PGR: 0.71, PLI: 0.7, SAC: 0.84). In the Confirmatory Factor Analysis, it shows a good fit of the data (CFI: 0.95, SRMR: 0.05, RMSEA: 0.04).

### 2.3. Data Analysis

When comparing scores between pairs of individuals in one of the dimensions of a questionnaire, the Euclidean differences can be the same, but the relative increase with respect to the overall construct in those scores may be different. Therefore, an alternative for comparing individuals is to work with the geometry proposed by Aitchison in 1986, where each dimension is understood as part of a construct whose sum is 100%.

The data analysis is carried out using the concepts and tools of Compositional Data Analysis, where each studied individual is represented as a vector of six components, each of which represents the proportion of the initial value of the dimension relative to the total scores of the individual. For example, the individual with ID number 643, whose initial values for each dimension were (24, 31, 13, 18, 13, 12), is transformed into a vector of normalized dimensions with values (4.80, 5.17, 2.60, 4.50, 2.60, 3.00) to give equal importance to each dimension. The sum of these normalized values is 22.67; dividing each value of the normalized vector by this sum yields the vector of proportions for this individual, which is (0.2118, 0.2279, 0.1147, 0.1985, 0.1147, 0.1324), where, by definition, the sum of the parts of this vector is 1, and each part represents the relative value (proportion expressed in part per unit) of this psychological dimension with respect to the total.

In the usual Euclidean space, to calculate the distance between two values, the difference between them is used, and the arithmetic mean is used to calculate an average, which is the sum of values divided by the number of values. In Compositional Data Analysis, the distance is defined as the difference between the logarithms (natural logarithms), known as “log-ratio”, and the indicator of centrality is not the arithmetic mean but the geometric mean, which is the nth root of the product of n values. For this reason, two measures of difference called “position ratios”, denoted as PR1 and PR2, are proposed for data analysis.

The first position ratio, PR1, involves calculating the log-ratio of each part of the vector of proportions and its geometric mean. Thus, for ID643, which serves as an example, we have:

gm(643) = (0.2118 · 0.2279 · 0.1147 · 0.1985 · 0.1147 · 0.1324)^1/6^ = 0.1599 RP1(643) = (0.2812, 0.3548, −0.3319, 0.2167, −0.3319, −0.1888)(1)

The interpretation of the PR1 vector provides a new perspective in the analysis of the individual’s PWB in relation to themselves because the sum of its parts is zero, meaning that positive and negative values balance each other. Positive values correspond to fractions greater than one, indicating a value above the individual’s mean, while negative values correspond to fractions less than one, indicating a value below the individual’s mean. This allows for a clearer interpretation of the relative position of each individual’s PWB dimensions.

The second position ratio, PR2, involves calculating the log-ratio of each part of the vector of proportions and the geometric mean of all individuals. In other words, it obtains an indicator of the dimension’s position in relation to the overall mean value in the considered group. Thus, for ID643, which serves as an example, we have:

gm(total) = (0.1748, 0.1508, 0.1527, 0.1814, 0.1672, 0.1634) PR2(643) = (0.1916, 0.4130, −0.2864, 0.0902, −0.3770, −0.2108)(2)

The PR2 position ratio offers a new perspective in the analysis of the individual’s PWB in relation to the considered group, allowing for a characterization relative to the group they belong to, considering social indicators that define the group (such as gender, age, profession, etc.). As before, positive values correspond to fractions greater than one, indicating a value above, in relative terms, the group’s mean, while negative values correspond to fractions less than one, indicating a value below, in relative terms, the group’s mean. This allows for the interpretation of the relative position of the PWB dimensions in relation to a certain pre-established group.

## 3. Results

In this section, the numerical results of the PWB data study from 628 individuals are presented according to the described methodology, accompanied by graphical illustrations to aid understanding and interpretation.

### 3.1. Study of Dimensional Proportions

**Figure 1:**
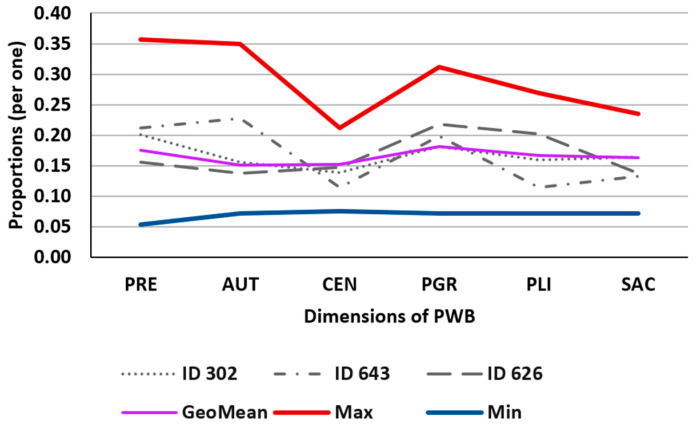
Illustration of the values in Table 1.

The proportions of PWB dimensions characterize each individual, and to interpret them, it is appropriate to compare their values with three position statistics: geometric mean, maximum, and minimum. Table 1 illustrates the values corresponding to three intentionally selected individuals to demonstrate the technique and mentioned statistics, as two have balanced profiles (302 with high scores and 626 with low scores) and the third one has an unbalanced profile (643). In Figure 1, the corresponding polygons representing these values are graphically depicted, providing a highly suitable and straightforward visual analysis of these values. The proportion values of individuals can be compared among themselves and in relation to the statistics.

For instance, when comparing individuals ID302 and ID626, both have markedly different direct scores in each dimension, yet their dimensions are balanced. ID302 has high scores for all dimensions (5.8, 4.5, 4.0, 5.2, 4.6, 4.75), while ID626 has lower scores (3.4, 3.0, 3.2, 4.75, 4.4, 3.0). When working with Compositional Data, we find that both individuals are very similar, meaning that the contribution of each dimension to psychological well-being is the same in both subjects. For individual ID643, the dimensions that contribute the most to well-being are PRE, AUT, and PGR, while the rest of the dimensions have a lesser weight in the construct. Therefore, the profiles of this individual are markedly different from those of ID302 and ID626, despite having similar direct scores (4.8, 5.17, 2.6, 4.5, 2.6, 3.0).

This allows us to conclude that ID302 and ID626 have very similar characteristics, yet at the same time, are different from ID643. Furthermore, ID302 and ID626 have more harmonious indicators (less difference between them), while ID643 exhibits much more pronounced relative differences.

### 3.2. Study of the Position Ratio PR1

**Figure 2:**
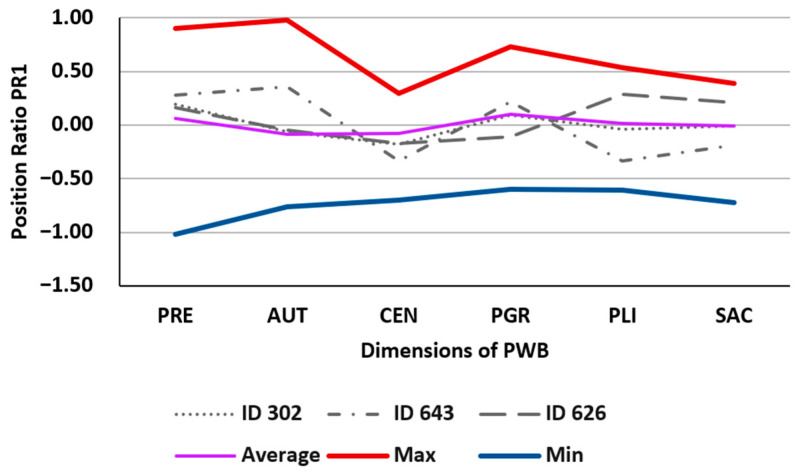
Illustration of the values in Table 2.

The PR1 position ratio characterizes the relative position (difference) of each proportion in relation to its central indicator (geometric mean). As before, it is appropriate to compare its value with three position statistics of PR1: arithmetic mean, maximum, and minimum. Table 2 illustrates the values corresponding to the same three previous individuals and the mentioned statistics. Figure 2 graphically represents the corresponding polygons.

For each individual, we can determine if each dimension of the PWB questionnaire contributes more or less to psychological well-being than the average of its dimensions by comparing each score with the zero axis. If the scores are positive, that dimension contributes more than the average, and if it is negative, it contributes less. For example, for ID643, the dimensions that contribute the most to their well-being are PRE, AUT, and PGR, while the rest contribute less; however, for subjects ID302 and ID626, all dimensions contribute equally to their well-being construct.

### 3.3. Study of the Position Ratio PR2

**Figure 3:**
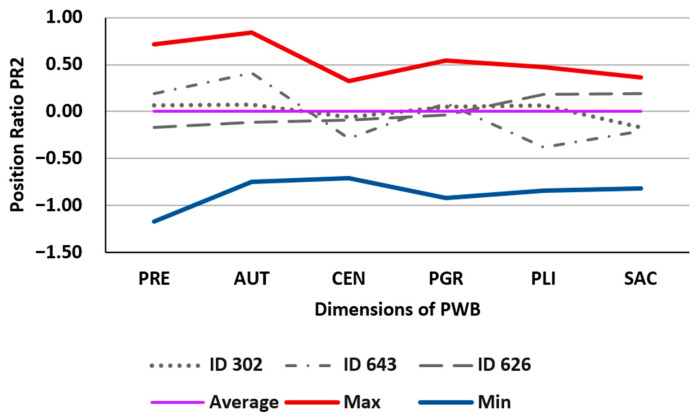
Illustration of the values in Table 3.

The PR2 position ratio characterizes the relative position (difference) of each proportion in relation to a central indicator of the specific dimension (geometric mean). As before, it is appropriate to compare its value with three position statistics of PR2: arithmetic mean, maximum, and minimum. Table 3 illustrates the values corresponding to the same three previous individuals and the mentioned statistics. Figure 3 graphically represents the corresponding polygons.

In this case, we can compare the scores of each dimension of the subjects with what each dimension contributes to the overall group of individuals, comparing it with the X-axis at 0. This allows us to determine if it is above or below the mean of the normative group. For example, we can observe that subject ID302 remains a very balanced individual in their scores, but when compared to the group, the SAC dimension is below the mean of the normative group. On the other hand, individual ID643, who is generally more imbalanced, has a PGR score very similar to the normative group, an AUT score much higher than the average, and a PLI score much lower.

### 3.4. Study of Subpopulations by Differential Characteristics

**Figure 4:**
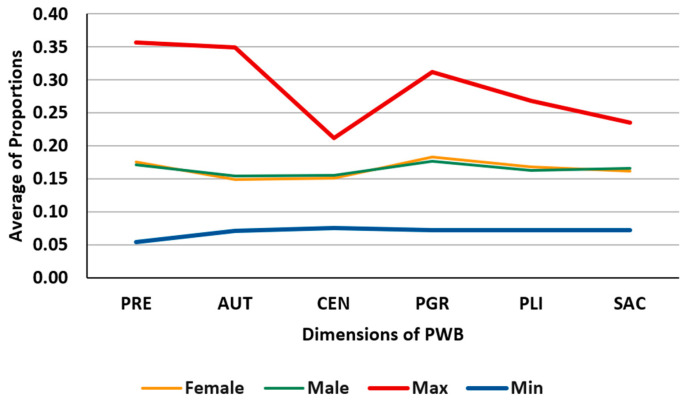
Illustration of the values in Table 4.

**Figure 5:**
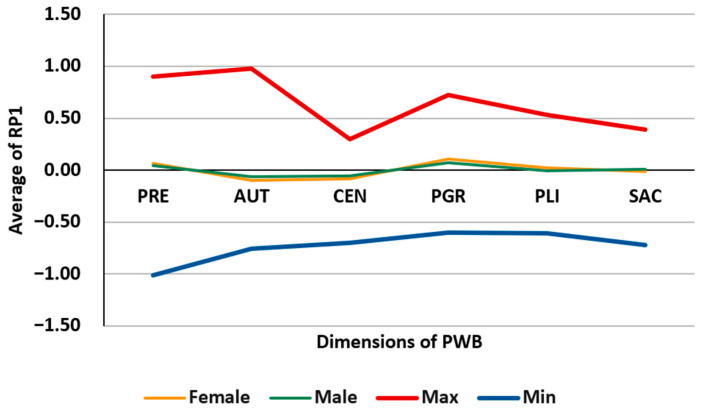
Illustration of the values in Table 5.

It may be of interest to analyze the dimensions of PWB in subpopulations of the sample defined by some assignable objective characteristic, such as gender, education level, age group, etc. Table 4 shows the mean values of the proportions in two groups: women and men; these values are illustrated along with the minimum and maximum statistics in Figure 4. Table 5 shows the mean values of the PR1 position ratios in the same two groups; these values are illustrated along with the minimum and maximum statistics in Figure 5. Both tables and figures indicate that there are no significant differences in the values of the dimensions corresponding to each of the two groups.

### 3.5. Proposal of a General Indicator of Subjective Psychological Well-Being (GISPW)

**Figure 6:**
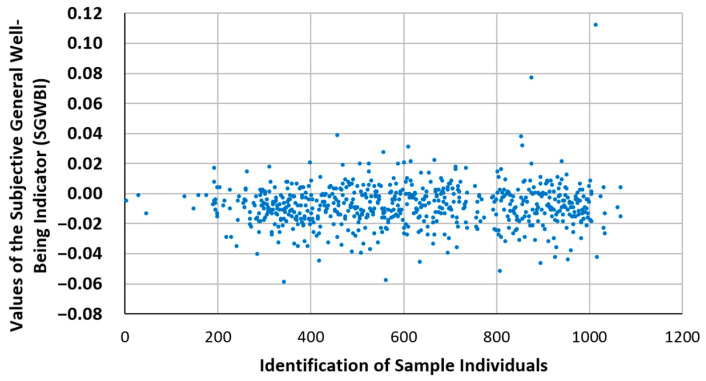
Illustration of the values of the GISPW in the studied population.

The concepts and tools of Compositional Data Analysis allow each individual to be characterized through position ratios that have been defined and formulated. This allows for a further step: defining a general indicator of subjective psychological well-being (GISPW), which undoubtedly constitutes the most original and relevant contribution of this article since, as far as we know, such an indicator neither exists nor has been defined in these analytical terms. The GISPW is defined as a weighted linear combination of the PR1 position ratios of each individual; the weighting is performed using weights with a sum of unity, which can be defined by the person conducting the psychological evaluation according to their professional judgment. Obviously, psychological balance corresponds to a GISPW value equal to zero, and positive or negative variations show imbalances relative to the central value. In Table 6, proposed values for the weights assigned to PWB dimensions for the calculation of the GISPW can be seen as an example. In Figure 6, the result of the GISPW calculation according to these weights is visualized for the sample of 628 individuals that serves as the framework for this work.

## 4. Discussion

The application of concepts and tools from Compositional Data Analysis provides a new perspective for quantitative analysis of PWB test results, which can enhance data interpretation and contribute to achieving the objectives of studies involving individuals. Until today, there have been no field studies in psychology, particularly in the field of psychological well-being, that have employed these statistical techniques.

In particular, this approach allows the individual to be considered as a whole and the study to be carried out through a systemic approach, where the contribution of each part is relevant both in itself and in relation to the total. The position ratios adequately express and highlight the values expressed in the PWB test. Prominent authors studying psychological well-being typically analyze the direct scores of each dimension and relate them to other constructs [19]; examine them in different individual situations, such as during the COVID-19 pandemic [20], based on whether they engage in exercise or not [21]; or consider their use of social media [22,23]. All of these are recent investigations on well-being. In particular, studies based on Carol Ryff’s questionnaire analyze sex differences [24,25] and psychophysiological variables [26,27], and compare them with other psychological variables, such as resilience [28,29], all using correlational studies and analyzing the scores obtained by individuals independently, rather than as part of the analyzed PWB construct with its six dimensions.

The proposed general indicator of subjective psychological well-being (GISPW) represents a quantitative and qualitative conceptual advancement, providing professionals with a new tool to better understand PWB test results as it offers novel information: the degree of balance among the scores. With this new indicator, for instance, it becomes possible to explore whether external factors correlate with PWB, but in a different way from traditional studies, as this proposal allows for the study of whether balance or imbalance among the dimensions correlates with other variables. In other words, as mentioned earlier, it is known that PWB correlates with resilience [28,29], but the question that this new indicator can answer is whether having a balanced pattern correlates with resilience, regardless of the direct score obtained in PWB. In our study, we have highlighted this by analyzing the patterns of three individuals: two of whom had very harmonious patterns, resulting in a GISPW index very close to zero, while another individual had a more irregular pattern, leading to a further deviation from zero.

A limitation of compositional data is that by themselves, they are insufficient in providing the total information, and their intention is to complement the information provided by the traditional way of correcting questionnaires.

It could be interesting to extend this study to both the general population and clinical samples to understand profiles in normative social as well as clinical groups.

This study innovatively demonstrates how profiles can be created for both individual and group cases regarding health. In subsequent research, it will be possible to investigate which profiles better predict physical and psychological health. This will help determine whether having a high score or balanced profiles is more important.

## 5. Conclusions

The analysis of psychological questionnaire data, particularly in this work with PWB from a Compositional Data Analysis perspective, provides relevant information for healthcare professionals to treat individuals in a more individualized manner.

The proposed general indicator GISPW helps to characterize patterns of psychological well-being that could assist healthcare professionals in understanding the balance or imbalance of individuals regarding their psychological well-being.

An interdisciplinary study such as the one presented in this article clearly demonstrates that quality is improved when a qualitative model is accompanied by an adequate quantitative model that allows for a better interpretation of the results according to the nature of the subject under study.

## Figures and Tables

**Table 1:** Values of the proportions of PWB dimensions for three selected individuals from the sample (ID302, ID643, ID626), and the overall geometric mean (GeoMean), absolute minimum (Min), and absolute maximum (Max). Source: own elaboration.

	PRE	AUT	CEN	PGR	PLI	SAC
ID302	0.2007	0.1557	0.1384	0.1817	0.1592	0.1644
ID643	0.2118	0.2279	0.1147	0.1985	0.1147	0.1324
ID626	0.1563	0.1379	0.1471	0.2184	0.2023	0.1379
GeoMean	0.1748	0.1508	0.1527	0.1814	0.1672	0.1634
Max	0.3571	0.3494	0.2120	0.3119	0.2686	0.2351
Min	0.0543	0.0717	0.0754	0.0723	0.0723	0.0723

Legends: PRE: Positive Relations with Others, AUT: Autonomy, CEN: Environmental Mastery, PGR: Personal Growth, PLI: Purpose in Life, and SAC: Self-acceptance.

**Table 2:** Values of the PR1 position ratio for the proportions of PWB dimensions for three selected individuals from the sample (ID302, ID643, ID626), and the overall mean value (Average), absolute maximum (Max) and absolute minimum (Min). Source: own elaboration.

PR1	PRE	AUT	CEN	PGR	PLI	SAC
ID302	0.1927	−0.0610	−0.1788	0.0931	−0.0391	−0.0070
ID643	0.2812	0.3548	−0.3319	0.2167	−0.3319	−0.1888
ID626	0.1638	−0.0469	−0.1720	−0.1075	0.2875	0.2110
Average	0.0596	−0.0881	−0.0754	0.0966	0.0152	−0.0080
Max	0.8992	0.9758	0.2964	0.7260	0.5328	0.3899
Min	−1.0157	−0.7606	−0.7009	−0.5997	−0.6082	−0.7210

Legends: PRE: Positive Relations with Others, AUT: Autonomy, CEN: Environmental Mastery, PGR: Personal Growth, PLI: Purpose in Life, and SAC: Self-acceptance.

**Table 3:** Values of the PR2 position ratio for the proportions of PWB dimensions for three randomly selected individuals from the sample (ID302, ID643, ID626), and the overall mean value (Average), absolute maximum (Max) and absolute minimum (Min). Source: own elaboration.

PR2	PRE	AUT	CEN	PGR	PLI	SAC
ID302	0.0650	0.0749	−0.0578	0.0505	0.0629	−0.1704
ID643	0.1916	0.4130	−0.2864	0.0902	−0.3770	−0.2108
ID626	−0.1720	−0.1119	−0.0893	−0.0375	0.1855	0.1903
Average	0.0000	0.0000	0.0000	0.0000	0.0000	0.0000
Max	0.7141	0.8401	0.3279	0.5419	0.4736	0.3639
Min	−1.1693	−0.7433	−0.7059	−0.9201	−0.8387	−0.8156

Legends: PRE: Positive Relations with Others, AUT: Autonomy, CEN: Environmental Mastery, PGR: Personal Growth, PLI: Purpose in Life, and SAC: Self-acceptance.

**Table 4:** Mean values of the proportions in men and women. Source: own elaboration.

	PRE	AUT	CEN	PGR	PLI	SAC
Female	0.1758	0.1496	0.1518	0.1830	0.1685	0.1624
Male	0.1718	0.1546	0.1557	0.1767	0.1635	0.1664
Max	0.3571	0.3494	0.2120	0.3119	0.2686	0.2351
Min	0.0543	0.0717	0.0754	0.0723	0.0723	0.0723

Legends: PRE: Positive Relations with Others, AUT: Autonomy, CEN: Environmental Mastery, PGR: Personal Growth, PLI: Purpose in Life, and SAC: Self-acceptance.

**Table 5:** Mean values of the PR1 position ratios in men and women. Source: own elaboration.

	PRE	AUT	CEN	PGR	PLI	SAC
Female	0.0652	−0.0966	−0.0821	0.1052	0.0226	−0.0143
Male	0.0431	−0.0628	−0.0555	0.0708	−0.0067	0.0111
Max	0.8992	0.9758	0.2964	0.7260	0.5328	0.3899
Min	−1.0157	−0.7606	−0.7009	−0.5997	−0.6082	−0.7210

Legends: PRE: Positive Relations with Others, AUT: Autonomy, CEN: Environmental Mastery, PGR: Personal Growth, PLI: Purpose in Life, and SAC: Self-acceptance.

**Table 6:** Values of the weights assigned to PWB dimensions for the calculation of the indicator of general subjective psychological well-being (GISPW). Source: own elaboration.

Dimension	PRE	AUT	CEN	PGR	PLI	SAC
Weight	0.18	0.22	0.18	0.12	0.18	0.12

Legends: PRE: Positive Relations with Others, AUT: Autonomy, CEN: Environmental Mastery, PGR: Personal Growth, PLI: Purpose in Life, and SAC: Self-acceptance.

## Data Availability

The data presented in this study are available on request from the first author.
